# Chronic Effects of Different Intensities of Power Training on Neuromuscular Parameters in Older People: A Systematic Review with Meta-analysis

**DOI:** 10.1186/s40798-023-00646-9

**Published:** 2023-10-24

**Authors:** Marcelo Bandeira-Guimarães, Eduarda Blanco-Rambo, Alexandra Ferreira Vieira, Mikel L. Sáez de Asteasu, Ronei S. Pinto, Mikel Izquierdo, Eduardo Lusa Cadore

**Affiliations:** 1 Exercise Research Laboratory (LAPEX), School of Physical Education, Physiotherapy and Dance, Universidade Federal do Rio Grande do Sul, Rua Felizardo 750, Bairro Jardim Botânico, Porto Alegre, Rio Grande do Sul CEP 90690-200 Brazil; 2 Navarrabiomed, Complejo Hospitalario de Navarra (CHN), Universidad Pública de Navarra (UPNA), IDISNA, Pamplona, Spain; 3 CIBER of Frailty and Healthy Aging (CIBERFES), Institute of Health Carlos III, Madrid, Spain

**Keywords:** Ageing, Muscle strength, Resistance training, Muscle power, Training intensity

## Abstract

**Background:**

Power training (PT) has been shown to be an effective method for improving muscle function, including maximal strength, measured by one-repetition maximum (1RM), and power output in older adults. However, it is not clear how PT intensity, expressed as a percentage of 1RM, affects the magnitude of these changes. The aim of this systematic review (International prospective register of systematic reviews—PROSPERO—registration: CRD42022369874) was to summarize the evidence from randomized clinical trials (RCT) assessing the effects of low-intensity (≤ 49% of 1RM) and moderate-intensity (50–69% of 1RM) versus high-intensity (≥ 70% of 1RM) PT on maximal power output and maximal strength in older adults.

**Methods:**

We included RCTs that examined the effects of different intensities of power training on maximum strength and power output in older people. The search was performed using PubMed, LILACS, Embase, and Scopus. Methodological quality was assessed using the preferred reporting items for systematic reviews and meta-analyses (PRISMA 2020 statement checklist), and the quality of evidence was determined using the PEDro scale. Data were analyzed using standardized mean differences (SMD) with a 95% confidence interval (CI), and random effects models were used for calculations. A significance level of *p* ≤ 0.05 was accepted.

**Results:**

Three RCTs assessing 179 participants, all of high methodological quality, were included. There were no significant differences between different PT intensities in terms of power output gains for leg press [SMD = 0.130 (95% CI − 0.19, 0.45), *p* = 0.425] and knee extension exercises [SMD: 0.016 (95% CI − 0.362, 0.395), *p* = 0.932], as well as leg press 1RM increases [SMD: 0.296 (95% CI − 0.03, 0.62); *p* = 0.072]. However, high-intensity PT (70–80% of 1RM) was significantly more effective than low-intensity PT in increasing 1RM for knee extension exercise [SMD: 0.523 (95% CI 0.14, 1.91), *p* = 0.008].

**Conclusions:**

PT performed at low-to-moderate intensities induces similar power gains compared to high-intensity PT (70–80% of 1RM) in older adults. Nonetheless, the influence of PT intensity on lower-limb strength gains seems to be dependent on the assessed exercise. Cautious interpretation is warranted considering the inclusion of only three studies.

**Supplementary Information:**

The online version contains supplementary material available at 10.1186/s40798-023-00646-9.

## Key Points

- Different intensities of power training (PT) promote similar increases in power output of leg press and knee extension exercises, as well as maximum strength of leg press exercise in older adults.
- High-intensity PT (70–80% of 1RM) seems to be more effective than low-intensity (20–40% of 1RM) PT in increasing maximum strength for knee extension.
- The results advance knowledge regarding the power training prescription, as they indicate that low- to moderate-intensity is a sufficient stimulus to promote marked improvements in mechanical muscle function in older adults.

**Supplementary Information:**

The online version contains supplementary material available at 10.1186/s40798-023-00646-9.

## Background

Power training, also known as explosive resistance training, is a type of resistance training that involves performing concentric muscle actions as fast as possible. This form of training has been widely recommended to improve physical functioning in older adults, as evidenced by several studies [1–3]. This recommendation has been applied not only to healthy older individuals [4–7], but also to those with chronic diseases such as diabetes mellitus and hypertension [8–11], geriatric syndromes [12–14], and even acutely hospitalized older patients [15, 16].

The rationale for prescribing power training in older adults is based on the following premises: first, muscle power output is more strongly associated with functional capacity than maximal strength and muscle size [17, 18]; second, muscle power output declines at a greater rate than maximal strength and muscle size during aging [19, 20]. Indeed, studies have shown that muscle power training induces superior gains in functional tests performance [21, 22], along with comparable gains in maximal strength and muscle hypertrophy [5] in older individuals.

Despite the recommendation for power training in older adults, the manipulation of power training variables (such as volume, intensity, and weekly frequency) to optimize its dose–response relationship has received less investigation compared to traditional resistance training variables. For instance, several meta-analyses have shown that maximal strength gains are optimized when resistance training intensity progresses to loads between 70 and 80% of one-repetition maximum (1RM) [23, 24]. However, research on power training intensity in older adults is limited, with few studies investigating the adaptations induced by different intensities (low- to moderate- vs. high-intensity) and showing no differences in maximal power output gains [25–27]. Regarding maximal strength gains, the results are controversial as one study observed an advantage in favor of high-intensity power training (i.e., 80% of 1RM) in single joint exercise, but no difference in multi-joint exercises was observed [27]. In addition, another study has shown that maximal strength increases with no differences between low and high-intensity power training, independently of the exercise [26].

Thus, there are controversial findings regarding the necessity of using higher intensities during power training in older adults to maximize mechanical muscle function gains. Therefore, a systematic review with meta-analysis is necessary to provide more solid evidence, considering these scarce and controversial findings in the literature. This study aims to summarize the evidence regarding the effects of different intensities of power training on maximal strength and maximal power output in older adults. We hypothesize that there will be no differences between low- to moderate-intensity versus high-intensity power training for muscle power output, but an advantage will be observed in favor of high-intensity power training for maximal strength.

## Methods

### Protocol and Registration

This research followed the preferred reporting items for systematic reviews and meta-analyses (PRISMA 2020 statement checklist) and was registered in the International Prospective Register of Systematic Reviews (PROSPERO, CRD42022369874) [28, 29].

### Eligibility Criteria

The eligibility criteria were based on the previously defined PICOT strategy (P, population: older adults; I, intervention: power training; C, comparator: different intensities of power training; O, outcomes: maximal strength gains, power output and/or muscle hypertrophy; T, type: randomized clinical trials). Thus, the eligible articles for this study were randomized clinical trials that compared the effects of at least two power training intensities, specifically high intensity (≥ 70% of 1RM) versus moderate (50–69% of 1RM) or low intensity (≤ 49% of 1RM). The interventions needed to include encouragement to participants to exert their maximal velocity during the concentric phase of each repetition. The study outcomes assessed included maximal dynamic strength, muscle power, and muscle hypertrophy in older individuals. Only manuscripts published in English language peer-reviewed journals were considered, and studies were included if they assessed older populations aged 60 years or older. Studies were excluded if they assessed individuals with any muscle injury or clinical condition that directly influenced the outcomes of interest.

### Search Strategy

The search was carried out in September of 2022 and updated in August 2023 using the electronic databases PubMed, LILACS, Embase, and Scopus. Additionally, manual searches of the references of the included studies were performed. There were no restrictions regarding the year of publication. The investigation comprised the following terms and MeSH terms (and their respective related terms): resistance training, power training, power-oriented resistance training, peak power, muscle performance, and hypertrophy. To optimize the capture of relevant references, such terms were combined by Boolean operators (OR and AND). Searches were delimited to the following fields: titles, descriptors, and abstract, and the selected descriptors should have been included in at least one of the three research fields. The full search strategy performed in the PubMed database is available in Additional file 1.

### Selection of Studies

The selection of studies was based on the eligibility criteria previously described, and each phase was carried out separately by two researchers and analyzed by a third reviewer as follows. First, two researchers independently evaluated the titles and abstracts of all studies found in the search (M.B-G. and E.B-R.). Studies with abstracts that did not provide sufficient information as per the inclusion and exclusion criteria were assessed separately in full. Subsequently, each study was assesses by the reviewers independently. Disagreements were resolved by consensus and, in cases of persistence, a third investigator adjudicated on the disagreement between the researchers (E.L.C.).

### Data Collection Process

Data extraction of each study selected was performed using a standardized form containing information on the methodological characteristics of the studies, participants (number of participants, sex, and age), interventions (intensity—% of 1RM, number of sessions and exercises) and outcomes (1RM and muscle power assessment). This process was performed independently by two researchers (M.B-G. and E.B-R.). Eventual disagreements were resolved by consensus or by a third reviewer (E.L.C.). When the studies did not present the data required for meta-analysis, the corresponding author was contacted. When the data were unavailable, the manuscript was excluded from the study. For data presented only graphically, the results were extracted using DigitizeIt®.

The extracted outcomes were the absolute deltas of the values referring to 1RM and muscle power. When not available, the delta was calculated from the values obtained before and after the intervention, and the delta standard deviation was imputed by the equation proposed by Higgins and Green [30].

### Risk of Bias Quality of Individual Studies

The risk of bias was assessed independently by two reviewers (M.B-G. and E.B-R.) using the PEDro scale based on the Delphi list, described by Verhagen et al. [31]. This procedure evaluates the risk of bias in the studies according to the following criteria: (1) eligibility criteria were specified; (2) participants were randomly allocated to groups; (3) allocation was concealed; (4) the groups were similar at baseline regarding the most important prognostic indicators; (5) there was blinding of all participants; (6) there was blinding of all therapists; (7) there was blinding of all assessors; (8) measures of at least one key outcome were obtained for more than 85% of the participants initially allocated to groups; (9) all participants for whom outcome measures were available received the treatment or control condition as allocated or, where this was not the case, data for at least one main outcome were analyzed by “intention to treat”; (10) the results of between-group statistical comparisons were reported for at least one main outcome; (11) the study provided both point measures and measures of variability for at least one main outcome. When these characteristics were described in the study, the criteria were considered met and the score was determined. Studies that did not describe these aspects did not score (Table 1). PEDro scores of 0–3 are considered “poor”, 4–5 “fair”, 6–8 “good”, and 9–10 “excellent” [32].

**Table 1:** PEDro scale for assessing the methodological quality of the included studies

Studies	Reid et al. [26]	Rodriguez-Lopez et al. [25]	de Vos et al. [27]
Eligibility criteria were specified	v	v	v
Participants were randomly allocated to groups	v	v	v
Allocation was concealed	x	x	x
The groups were similar at baseline	v	v	v
There was blinding of all participants	v	x	v
There was blinding of all assessors	v	x	x
There was blinding of all therapists	x	x	x
At least one key outcome was obtained for more than 85% of the participants	v	v	v
Treatment or control regarding allocation or intention to treat	v	v	v
The results of between-group statistical comparisons were reported for at least one key outcome	v	v	v
The study provided both point measures and measures of variability for at least one key outcome	v	v	v
Total	8	6	7

v: Reported in the study; x: Not reported in the study

### Data Synthesis and Analysis

Data analysis involved a meta-analysis comparing the effects of high-intensity power training (≥ 70%1RM) versus moderate (50–69% of 1RM) or low intensity (≤ 49%1RM) on the neuromuscular parameters of older individuals. The results are presented as standardized mean differences (SMD) for 1RM and maximal power output with 95% confidence intervals (CI). Calculations were performed using random effects models. The I^2^ inconsistency test was used to assess the statistical heterogeneity of treatment effects between studies, with values higher than 50% indicating high heterogeneity [31]. Values of α ≤ 0.05 were considered statistically significant.

To explore between-study heterogeneity further, we employed the “leave-one-out” strategy, where studies were removed individually from each meta-analysis. This exploratory procedure is recommended, particularly for meta-analyses with a limited number of studies [33]. We only considered analyses that included at least two studies in all comparisons.

We verified publication bias by visually inspecting the funnel plot for each analyzed variable. Asymmetry was tested using the Begg and Egger test, with significance set at *p* = 0.010. When publication bias was detected, we used the trim-and-fill test to estimate its effect on interpreting the results; however, when this was necessary the results were unchanged after the test. We performed all analyses using Stata version 15.1.

## Results

### Study Selection

**Fig. 1:**
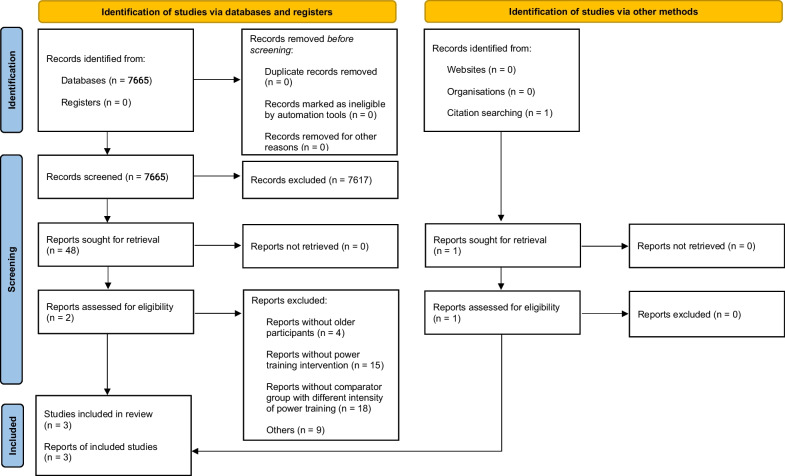
PRISMA 2020 flow diagram of included studies. From: Page MJ, McKenzie JE, Bossuyt PM, Boutron I, Hoffmann TC, Mulrow CD, et al. The PRISMA 2020 statement: an updated guideline for reporting systematic reviews. BMJ 2021;372:n71. https://doi.org/10.1136/bmj.n71

A search on the PubMed (Medline), LILACS, Embase and Scopus databases yielded a total of 7665 references, and an additional one study was identified through manual searches. After removing duplicates and reviewing titles, 7617 studies were excluded. Forty-eight articles were further examined by reading the abstracts, resulting in the exclusion of 46 articles. Upon a thorough reading of the two remaining studies and one additional study identified through manual searches, three studies met the inclusion criteria and were included in the quantitative analysis (see Fig. 1). While our study initially aimed to evaluate muscle power, maximal strength, and muscle hypertrophy, we found only one study that met the inclusion criteria for assessing muscle hypertrophy in older individuals [25]. Therefore, we were unable to conduct a meta-analysis for this outcome.

### Study Characteristics

The general characteristics of selected studies are described in Table 2.

**Table 2:** General characteristics of included studies

Study	Intervention	Sample	Sex (M/F)	Mean age ± SD	Period (weeks)	Total sessions	Intensities	Sets versus repetitions	Exercises	Outcomes
Reid et al. [26]	LI	25	10/15	78 ± 5	16	32	40%1RM	3 × 10 (40%1RM)	Bilateral leg press Unilateral knee extension	1RM Peak power
	HI	27	9/18	77.6 ± 4	16	32	70%1RM	3 × 10 (70%1RM)		
Rodriguez-Lopez et al. [25]	LI	21	9/12	70.6	12	24	40%1RM	6 × 12 (40%1RM)	Unilateral leg press Knee extension	1RM Peak power
	HI	22	10/12	70.6	12	24	80%1RM	6 × 6 (80%1RM)		
De Vos et al. [27]	LI	28	11/17	69.4 ± 5.8	8–12	16–24	20%1RM	d1: 3 × 8 (20%1RM) d2: 1RM test + 2 × 8 (20%1RM)	Leg press Chest press Leg extension Seated row Leg flexion	1RM Peak power
	MI	28	11/17	68.1 ± 4.5	8–12	16–24	50%1RM	d1: 3 × 8 (50%1RM) d2: 1RM test + 2 × 8 (50%1RM)		
	HI	28	11/17	69 ± 6.4	8–12	16–24	80%1RM	d1: 3 × 8 (80%1RM) d2: 1RM test + 2 × 8 (80%1RM)		

*LI* low-intensity group, *MI* moderate-intensity group, *HI* high-intensity group, *1RM* one-repetition maximum, *d1* day one (Tuesday); *d2* day two (Thursday)

#### Description of the Participants

The three studies included in this meta-analysis assessed a total of 179 individuals, consisting of 71 men and 108 women. Participants' ages ranged from 63 to 83 years, and the sample sizes ranged from 43 to 84 participants [25–27]. All studies reported obtaining written informed consent from all participants before the start of the study, and all training sessions were supervised. The authors also reported a total of six exercise-related adverse events: one hamstring injury and back pain after a training session; and, a non-injurious fall outside of the laboratory after a training visit in study by Reid et al. [26]; three joint and musculoskeletal pain and one inguinal hernia in study by De Vos et al. [27]. These adverse events led to six withdrawals related to the training protocol: two in study by Reid et al. [26]; four in study by De Vos et al. [27]. Twenty six participants withdrew from the studies for reasons unrelated to the studies’ protocol [25–27].

#### Outcomes Assessment

Among the included studies, maximal strength was assessed by the 1RM test in the leg press and knee extension [25–27]. Concerning power output, both Reid et al. [26] and De Vos et al. [27] utilized pneumatic strength training equipment to assess peak power in the leg press and knee extension exercises. Rodriguez-Lopez et al. [25] utilized a linear position transducer and a force plate to assess peak power in the leg press exercise, and a custom-built rigid chair instrumented with a strain gauge to assess rate of force development in knee extension exercise.

#### Description of the Interventions

The studies by Rodriguez-Lopez et al. [25] and De Vos et al. [27] assessed a non-exercising control group; however, these control groups were not used in our analysis. Two studies conducted their interventions and assessments on lower-limbs exercises [25, 26], and one study combined both lower- and upper-limbs exercises [27].

Additional file 1: Tables S1 and S2 provide a summary of the study results, categorized by the assessed exercise (i.e., leg press and knee extension). All studies used fixed intensities throughout their interventions [25–27]. The intensities compared in the interventions were as follows: 20%, 50%, and 80% of 1RM [27], 40% and 80% of 1RM [25], and 40% and 70% of 1RM [26].

Two studies used equal volume (sets and repetitions) to compare the groups throughout the interventions: one prescribed three sets of 10 repetitions [26], and the other prescribed three sets of eight repetitions on the first training session (Tuesdays) and two sets of eight repetitions on the second session (Thursdays) of the week [27]. Rodriguez-Lopez et al. [25] prescribed different volumes (sets) for low- and high-intensity power training groups, but equal total volume (number of repetitions × external load relative to 1-RM): the low-intensity group performed six sets of twelve repetitions with a load equivalent to 40% 1-RM, while the high-intensity group performed six sets of six repetitions with a load equivalent to 80% 1-RM.

The weekly training frequency was 2 times a week in all included studies, while the intervention period ranged from 8 to 16 weeks. The total number of training sessions ranged from 16 to 32 across studies. All included articles evaluated 1RM on knee extension and leg press exercises before and after training [25–27], as well all studies assessed maximal power output on knee extension and leg press exercises [25–27].

#### Risk of Bias

Among the included studies, three (100%) specified their eligibility criteria and reported that participants were randomly allocated to groups and the groups were similar at baseline. Two articles (66.6%) blinded the participants, one (33.3%) blinded the assessors and none blinded the therapists (0%). In addition, all three studies (100%) obtained measures of at least one key outcome from more than 85% of the participants, performed treatment or control regarding allocation or intention to treat, compared between-group statistics, and presented point measures and measures of variability for at least one main outcome (Table 1).

#### Effects of Interventions

Table 3 presents a summary of the meta-analysis results. One of the studies we selected [27] compared three different power training intensities: low-intensity (20%1RM), moderate-intensity (50%1RM), and high-intensity (80%1RM). Therefore, for each exercise and outcome (maximal strength and power output), we conducted two separate analyses. One analysis compared low- versus high-intensity from the study by De Vos et al. [27], while the other analysis compared moderate- versus high-intensity from the same study, along with the other studies [25, 26].

**Table 3:** Summary of meta-analysis results

Outcome measure	SMD (95% CI)	Z value	*p* value	I^2^ (%)
Leg press 1RM^a^	0.296	1.800	0.072	11.4
Leg press 1RM^b^	0.266	1.621	0.105	28.2
Knee extension 1RM^a^	0.523	2.662	0.008*	0.0
Knee extension 1RM^b^	0.286	1.477	0.140	0.0
Leg press power^a^	0.130	0.799	0.425	0.0
Leg press power^b^	0.065	0.395	0.693	0.0
Knee extension power^a^	0.016	0.085	0.932	40.3
Knee extension power^b^	− 0.134	− 0.693	0.488	0.0

*1RM* one-repetition maximum, *SMD* standardized mean difference, *I*^*2*^ heterogeneity

^a^Low- (20–40%1RM) versus high-intensity (70–80% of 1RM) (comparison using the group training at 20% of 1RM from study by De Vos et al. [27])

^b^low-to-moderate- (40–50%1RM) versus High-intensity (70–80% of 1RM) (comparison using the group training at 50% of 1RM from study by De Vos et al. [27])

**Fig. 2:**
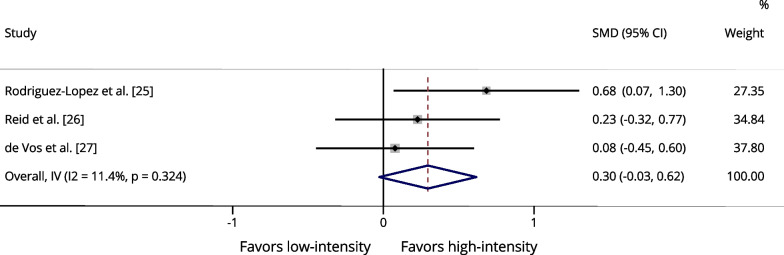
Forest plot of the effects of low-intensity (20–40% of 1RM) power training versus high-intensity (70–80% of 1RM) power training on the maximum strength assessed by leg press 1RM. The squares and error bars signify the SMDs and 95% CI values; the diamonds represent the pooled estimates of random effects meta-analyses. *SMDs* standardized mean differences, *CI* confidence interval

**Fig. 3:**
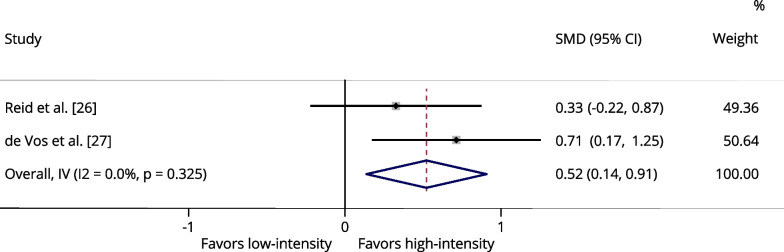
Forest plot of the effects of low-intensity (20–40%% of 1RM) power training versus high-intensity (70–80% of 1RM) power training on the maximum strength assessed by knee extension exercise 1RM. The squares and error bars signify the SMDs and 95% CI values; the diamonds represent the pooled estimates of random effects meta-analyses. *SMDs* standardized mean differences, *CI* confidence interval

The maximum strength data for leg press were assessed in three studies [25–27] and included 179 participants. There were no significant differences between low and high-intensity power training for leg press 1RM (Fig. 2), as well as there was no significant difference between low- to moderate-intensity and high-intensity power training for leg press 1RM (Additional file 1: Fig. S1). The maximum strength data for knee extension were assessed in two studies [26, 27] and included 136 participants. No significant difference was found between low- to moderate-intensity and high-intensity power training for knee extension 1RM (Additional file 1: Fig. S2). However, high-intensity power training was significantly associated with greater strength gains for knee extension 1RM compared to low-intensity (*p* = 0.008 [I^2^: 0.0%]) (Fig. 3).

**Fig. 4:**
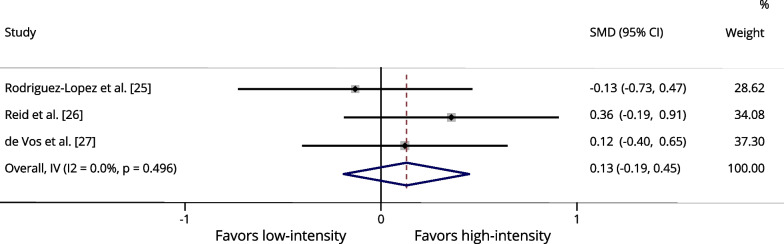
Forest plot of the effects of low-intensity (20–40% of 1RM) power training versus high-intensity (70–80% of 1RM) power training on the power output assessed in the leg press exercise. The squares and error bars signify the SMDs and 95% CI values; the diamonds represent the pooled estimates of random effects meta-analyses. *SMDs* standardized mean differences, *CI* confidence interval

**Fig. 5:**
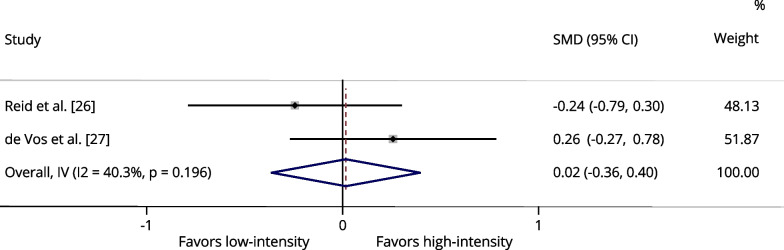
Forest plot of the effects of low-intensity (20–40% of 1RM) power training versus high-intensity (70–80% of 1RM) power training on the power output assessed in the knee extension exercise. The squares and error bars signify the SMDs and 95% CI values; the diamonds represent the pooled estimates of random effects meta-analyses. *SMDs* standardized mean differences, *CI* confidence interval

Regarding muscle power output, three studies that assessed leg press exercises were analyzed, comprising 179 participants [25–27]. There were no significant differences between low-intensity and low- to moderate-intensity compared to high-intensity power training on this outcome [*p* = 0.425 (I^2^: 0.0%); and, *p* = 0.693 (I^2^: 0.0%), respectively] (Fig. 4 and Additional file 1: Fig. S3, respectively). Regarding the knee extension exercise, two studies that assessed muscle power output were analyzed, comprising 136 participants [26, 27]. There were no significant differences between low-intensity and low- to moderate-intensity compared to high-intensity power training on this outcome [*p* = 0.932 (I^2^: 40.3%); and, *p* = 0.488 (I^2^: 0.0%), respectively] (Fig. 5 and Additional file 1: Fig. S4, respectively).

## Discussion

The purpose of this systematic review with meta-analysis was to summarize the evidence on the effects of low- to moderate-intensity versus high-intensity power training on maximal strength and power output in older adults. Unfortunately, due to the limited number of studies available, it was not possible to conduct a quantitative analysis of muscle hypertrophy. The main findings of the present review indicate that both low- to moderate-intensity (20–50% of 1RM) and high-intensity (70–80% of 1RM) power training induce similar gains in maximal power output. Additionally, the power training intensities analyzed induced comparable maximal strength gains in the leg press exercise, but higher intensities resulted in greater strength increases than low-intensity in the knee extension exercise.

All studies included in the present review found similar increases in maximal power output, regardless of the power training intensity [25–27]. In particular, the study by De Vos et al. [27] observed similar muscle power gains when comparing three different intensities (80% vs. 50% vs. 20% of 1RM). These findings are consistent with a systematic review by Straight et al. [34], which concluded that power training is an effective intervention for improving muscle power. In addition, in a previous systematic review with meta-analysis, Byrne et al. [35] have demonstrated the efficacy of different methods of power training interventions on muscle power gains. These results are important because muscle power is strongly associated with functional capacity, even more than maximal strength and muscle size [3, 34, 36, 37]. Muscle power also declines at a faster rate with age than maximal strength and muscle size [3, 20]. The gains induced by power training are associated with neural adaptations, such as increases in maximal motor unit recruitment capacity, maximal firing rate, and motor unit double discharges [19]. Nevertheless, no previous systematic review with meta-analysis has summarized the evidence on the effects of different intensities of power training on muscle function outcomes in older adults.

From a practical perspective, it is not necessary for older adults to exercise at high power training intensities (i.e., ≥ 70% of 1RM) to achieve gains in power output. Those who are not able to exercise at higher intensities due to clinical conditions, such as frail older adults and those with multiple comorbidities, may still achieve improvements by training at low- to moderate-intensities (20–50% of 1RM in the included studies). In fact, previous studies have demonstrated that low- to moderate-intensity training can promote several benefits, including muscle power gains in mobility-limited [26] and physically frail older individuals [12, 14, 16]. Furthermore, because training at higher intensities is associated with greater pain and discomfort post-exercise [38], training at lower intensities may reduce pain and discomfort, thus improving intervention adherence [39].

In our analysis of maximal strength gains (measured by 1RM) following power training, we found that higher intensities did not necessarily lead to greater gains in the leg press exercise. However, although there was no significant difference comparing low- to moderate-intensity (i.e., 40–50% of 1RM) versus high-intensity power training in the knee extension 1RM, we observed significant differences in strength gains for knee extension comparing low-intensity (20–40% of 1RM) versus high-intensity (SMD = 0.523), suggesting that the influence of relative intensity on maximal strength gains seems to be exercise-dependent.

Individual studies also showed significant improvements in maximal strength gains following different intensities [25, 27]. Rodriguez-Lopez et al. [25] found a significant difference in leg press 1RM between groups performing power training at 40% versus 80% of 1RM after 12 weeks. De Vos et al. [27] observed greater 1RM gains for several exercises (including knee extension) when training at 80% versus 20% of 1RM, while a significant difference in favor of high-intensity was observed only in the seated row exercise when comparing 80% versus 50% of 1RM.

Interestingly, our findings are consistent with those of traditional resistance training studies, in which greater maximal strength gains are observed when the intensity progresses up to 70–80% of 1RM [23, 24] for knee extension exercise. However, for the leg press exercise, we found that power training at intensities up to 50% of 1RM can be effective in promoting maximal strength gains. This finding has an interesting practical application, as the leg press exercise involves similar muscle groups to functional tasks like sit-to-stand and climbing stairs.

The reason for the different findings between exercises is not entirely clear, but one possible explanation is the characteristic of motor unit (MU) recruitment during power training. The threshold of muscle force at which MUs are recruited (i.e. recruitment threshold) is lower at high speed of muscle contraction [40], making it possible for type II MUs to be recruited even at low to moderate loading intensities. Since the recruitment of type II MUs is crucial for inducing maximal strength gains, this could explain the lack of difference in maximal strength gains between lower and higher intensities for the leg press exercise.

An important issue that needs to be mentioned is that, among the few studies included in our systematic review with meta-analysis, only one study used moderate intensity (i.e., 50% of 1RM), which is lower than the intensity used in several studies that have reported marked strength gains using this type of intervention (i.e., progressing to 60% of 1RM) [5, 7, 12, 14, 16]. Therefore, more studies are needed to compare low- versus high-intensities, and mainly moderate- versus high-intensities, and to assess the strength gains from different exercises to determine if low- to moderate-intensities are sufficient to optimize strength gains during power training.

### Limitations and Strengths

This systematic review presents some limitations that should be acknowledged. The small number of included studies did not allow for sensitivity analyses. Furthermore, only one study analyzed the effects of different power training intensities in upper body exercises, and our results cannot be extrapolated to these exercises. Additionally, the included studies only assessed older participants without diseases that could directly interfere with outcome measures. Therefore, our findings cannot be extrapolated to older individuals with different clinical conditions (e.g., geriatric syndromes, musculoskeletal injuries, chronic conditions). It is important to note that the authors of the present systematic review were unable to control for these limitations.

However, it is worth noting that this review implemented a rigorous process that adhered to recommended practices in systematic reviews. This process included two independent researchers in all stages of study selection and data extraction. Additionally, the methodological quality of the included studies assessed by the PEDro scale was classified as “good” (i.e., low risk of bias). Moreover, to the best of our knowledge, this is the first systematic review with a meta-analysis assessing the effects of different power training intensities on neuromuscular adaptations in older adults.

## Conclusions

In summary, power training performed at low-intensity (20–40% of 1RM), low-to-moderate intensity (40–50% of 1RM) and high-intensity (70–80% of 1RM) induces similar muscle power output gains in older adults. Additionally, these intensities also promote comparable maximal strength gains in the leg press exercise, but there is an advantage in favor of high-intensity compared to low-intensity power training in the knee extension exercise. These findings advance knowledge regarding the power training prescription, as they indicate that low- to moderate-intensity is a sufficient stimulus to promote marked improvements in mechanical muscle function in older adults. From a practical standpoint, considering the findings summarized by the present systematic review, exercise professionals dealing with power training prescription for older adults may optimize muscle power output gains as well as maximal strength from multi-joint lower-body exercises using low to moderate intensities (40–50% of 1RM), while higher intensities (70–80% 1RM) may be advantageous to develop maximal strength in single-joint lower-body exercise compared to low intensity power training (20–40% of 1RM). Nevertheless, considering the few studies were found in our search, further randomized clinical trials comparing different intensities of power training with different exercises should be designed to enhance the strength of the evidence.

### Supplementary Information

**Additional file 1.** Search strategy, additional table and figures.

## Data Availability

The datasets used and/or analyzed during the current study are available from the corresponding author on reasonable request.

## Abbreviations

PT: Power training
1RM: One-repetition maximum
PROSPERO: International prospective register of systematic reviews
SMD: Standardized mean differences
CI: Confidence interval
PRISMA: Preferred reporting items for systematic reviews and meta-analyses
MU: Motor unit
