# Supplementary material for: Chronic Effects of Different Intensities of Power Training on Neuromuscular Parameters in Older People: A Systematic Review with Meta-analysis
Source: Sports Med Open. 2023 Oct 24;9:98. doi: 10.1186/s40798-023-00646-9 (PMC10597949; doi:10.1186/s40798-023-00646-9)
Supplement: Supplementary file 1 — Additional file 1. Search strategy, additional table and figures. [file 40798_2023_646_MOESM1_ESM.docx]

**Search Strategy**

*(((((((((((((((((((((((((resistance training[Title/Abstract]) OR (power training[Title/Abstract])) OR (high-speed resistance training[Title/Abstract])) OR (high-velocity resistance training[Title/Abstract])) OR (power-oriented resistance training[Title/Abstract])) AND (peak power[Title/Abstract])) OR (muscle performance[Title/Abstract])) OR (neuromuscular performance[Title/Abstract])) OR (maximum strength[Title/Abstract])) OR (muscle strength[Title/Abstract])) OR (one-repetition maximum[Title/Abstract])) OR (one repetition maximum[Title/Abstract])) OR (hypertrophy[Title/Abstract])) OR (muscle hypertrophy[Title/Abstract])) OR (muscle power[Title/Abstract])) OR (velocity[Title/Abstract])) OR (sprint[Title/Abstract])) OR (jump[Title/Abstract])) OR (vertical jump[Title/Abstract])) OR (horizontal jump[Title/Abstract])) OR (squat jump[Title/Abstract])) OR (squat-jump[Title/Abstract])) OR (countermovement jump[Title/Abstract])) OR (power output[Title/Abstract])) OR (average power[Title/Abstract])) OR (maximal power[Title/Abstract])*

Filters applied in PubMed database: “*Full text*”, “*Randomized Controlled Trial*”, “*English*”, “*Aged: 65+ years*”, “*Middle Aged: 45 - 64 years*”. The Pubmed search results are presented in Figure S5.

**Table S1**: Summary of the results of the included studies assessing leg press exercise.

| **Study** | **Group** | **1RM**  **Pre**  **Mean ± SD** | **1RM**  **Post**  **Mean ± SD** | **1RM**  **Time effect** | **Power**  **Pre**  **Mean ± SD** | **Power**  **Post**  **Mean ± SD** | **Power**  **Time effect** |
| --- | --- | --- | --- | --- | --- | --- | --- |
| Reid et al. [26] | LI | 882 ± 258 N | 13.3 ± 4% (SE) | p < 0.001 | 273 ± 131 W | 32.9 ± 13% (SE) | p < 0.01 |
|  | HI | 940 ± 344 N | 19.2 ± 4% (SE) | p < 0.0001 | 282 ± 153 W | 41.6 ± 12% (SE) | p < 0.01 |
| Rodriguez-Lopez et al. [25] | LI | 71.2 ± 20.8 kg | 11.7 ± 7.6% | p ≤ 0.001 | 164 ± 81 W | 66 ± 70% | p ≤ 0.001 |
|  | HI | 70.7 ± 22.4 kg | 16.8 ± 7.3% | p ≤ 0.001 | 189 ± 97 W | 57 ± 67% | p ≤ 0.001 |
| de Vos et al. [27] | LI | 1057 ± 395 N | 1203 ± 48 N | p < 0.0001 | 611 ± 219 W | 662 ± 291 W | p < 0.0001 |
|  | MI | 1121 ± 363 N | 1290 ± 505 N | p < 0.0001 | 671 ± 246 W | 756 ± 306 W | p < 0.0001 |
|  | HI | 1190 ± 432 N | 1359 ± 548 N | p < 0.0001 | 668 ± 259 W | 737 ± 312 W | p < 0.0001 |

LI: low-intensity group; MI: moderate-intensity group; HI: high-intensity group; 1RM: one-repetition maximum; SD: Standard deviation; (SE): Standard error.

**Table S2**: Summary of the results of the included studies assessing knee extension exercise.

| **Studies** | **Groups** | **1RM**  **Pre**  **Mean ± SD** | **1RM**  **Post**  **Mean ± SD** | **1RM**  **Time effect** | **Power**  **Pre**  **Mean ± SD** | **Power**  **Post**  **Mean ± SD** | **Power**  **Time effect** |
| --- | --- | --- | --- | --- | --- | --- | --- |
| Reid et al. [26] | LI | 58.1 ± 24 N | 20.3 ± 5 % (SE) | p < 0.01 | 61.6 ± 28 W | 27.1 ± 8% (SE) | p < 0.01 |
|  | HI | 65.7 ± 25 N | 22.4 ± 7 % (SE) | p < 0.01 | 76.7 ± 38 W | 27.6 ± 12% (SE) | p < 0.05 |
| de Vos et al. [27] | LI | 124 ± 46 Nm | 143 ± 62 Nm | p < 0.0001 | 264 ± 118 W | 294 ± 143 W | p < 0.0001 |
|  | MI | 139 ± 45 Nm | 169 ± 62 Nm | p < 0.0001 | 302 ± 123 W | 349 ± 168 W | p < 0.0001 |
|  | HI | 141 ± 53 Nm | 177 ± 77 Nm | p < 0.0001 | 290 ± 128 W | 332 ± 162 W | p < 0.0001 |

LI: low-intensity group; MI: moderate-intensity group; HI: high-intensity group; 1RM: one-repetition maximum; SD: Standard deviation; (SE): Standard Error.


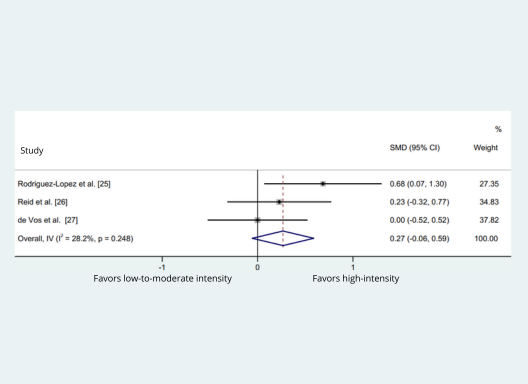


**Figure S1:** Forest plot comparing the effects of low- to moderate-intensity (40-50% 1RM) and high-intensity (70 - 80% 1RM) power training in maximum strength gains of leg press exercise. The squares and error bars signify the SMDs and 95% CI values; the diamonds represent the pooled estimates of random effects meta-analyses. SMDs = standardized mean differences; CI = confidence interval.


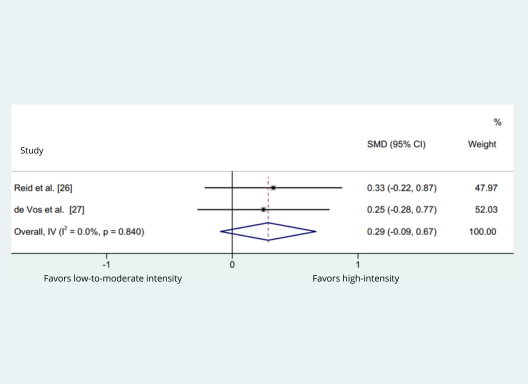


**Figure S2:** Forest plot comparing the effects of low- to moderate-intensity (40-50% 1RM) and high-intensity (70 - 80% 1RM) power training in maximum strength gains of knee extension exercise. The squares and error bars signify the SMDs and 95% CI values; the diamonds represent the pooled estimates of random effects meta-analyses. SMDs = standardized mean differences; CI = confidence interval.


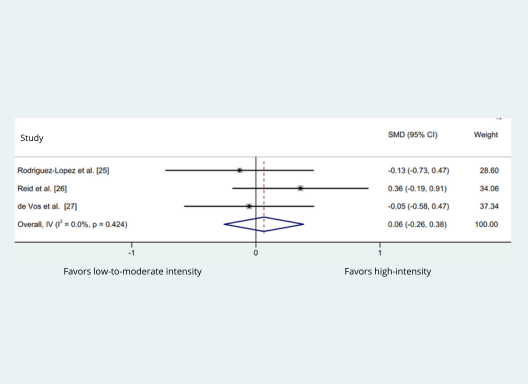


**Figure S3:** Forest plot comparing the effects of low- to moderate-intensity (40-50% 1RM) and high-intensity (70 - 80% 1RM) power training in power output gains of leg press exercise. The squares and error bars signify the SMDs and 95% CI values; the diamonds represent the pooled estimates of random effects meta-analyses. SMDs = standardized mean differences; CI = confidence interval.


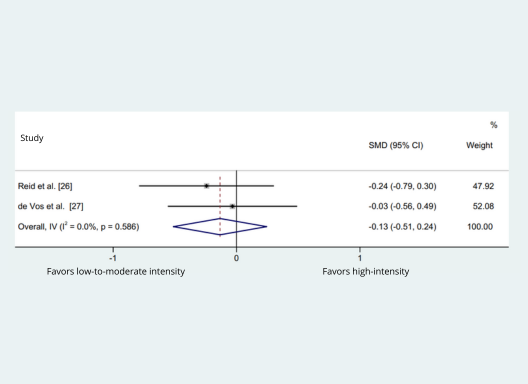


**Figure S4:** Forest plot comparing the effects of low- to moderate-intensity (40-50% 1RM) and high-intensity (70-80% 1RM) power training in power output gains of knee extension exercise. The squares and error bars signify the SMDs and 95% CI values; the diamonds represent the pooled estimates of random effects meta-analyses. SMDs = standardized mean differences; CI = confidence interval.


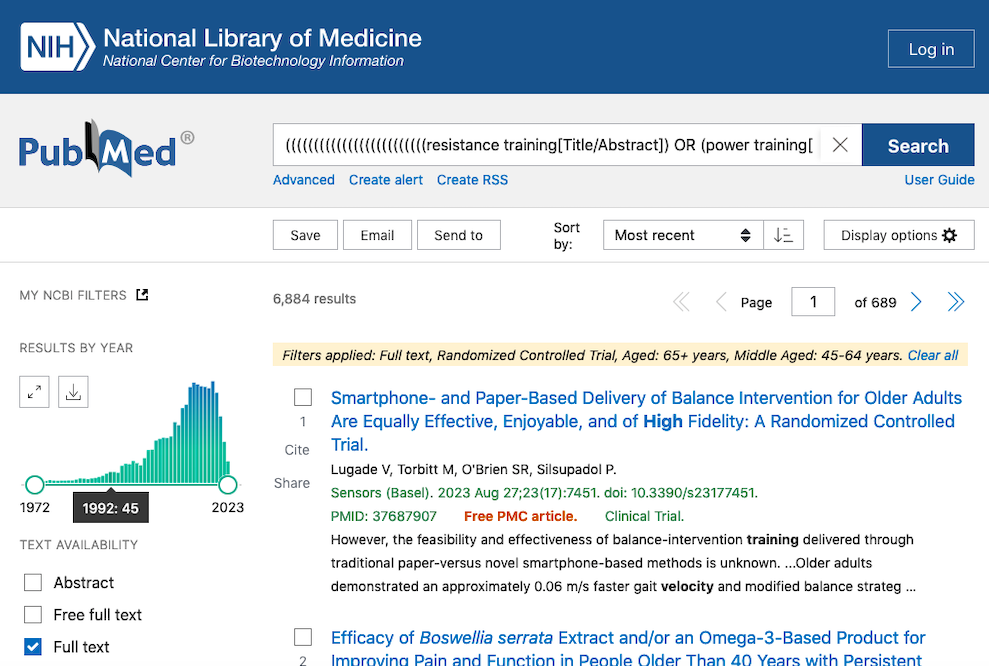


Figure S5: “Print screen” of the Pubmed search results.
